# Implementation of Complete Boolean Logic Functions in Single Complementary Resistive Switch

**DOI:** 10.1038/srep15467

**Published:** 2015-10-21

**Authors:** Shuang Gao, Fei Zeng, Minjuan Wang, Guangyue Wang, Cheng Song, Feng Pan

**Affiliations:** 1 Key Laboratory of Advanced Materials (MOE), School of Materials Science and Engineering, Tsinghua University, Beijing, 100084, China

## Abstract

The unique complementary switching behaviour of complementary resistive switches (CRSs) makes them very attractive for logic applications. The implementation of complete Boolean logic functions in a single CRS cell is certainly an extremely important step towards the commercialisation of related logic circuits, but it has not been accomplished to date. Here, we report two methods for the implementation of complete Boolean logic functions in a single CRS cell. The first method is based on the intrinsic switchable diode of a peculiar CRS cell that is composed of two anti-serial bipolar resistive switches with a rectifying high resistance state, while the second method is based directly on the complementary switching behaviour itself of any single CRS cell. The feasibilities of both methods have been theoretically predicted and then experimentally demonstrated on the basis of a Ta/Ta_2_O_5_/Pt/Ta_2_O_5_/Ta CRS cell. Therefore, these two methods—in particular the complementary switching behaviour itself-based method, which has natural immunity to the sneak-path issue of crossbar logic circuits—are believed to be capable of significantly advancing both our understanding and commercialization of related logic circuits. Moreover, peculiar CRS cells have been demonstrated to be feasible for tri-level storage, which can serve as an alternative method of realising ultra-high-density data storage.

Logic circuits based on conventional CMOS technology are the basic building blocks of real-world computers. The continued downscaling of conventional logic circuits according to Moore’s law has contributed crucially to the prosperity of today’s information era, but it is now approaching its physical limit mainly due to the significant increase in leakage current^1^. To extend Moore’s law, resistive random access memories (RRAMs), which are acknowledged as one of the most promising candidates for the state-of-the-art nonvolatile memories (i.e., silicon-based Flash memories)^2^,^3^,^4^,^5^,^6^, have recently been introduced to the research field of logic circuits^2^,^4^. Compared with conventional CMOS logic circuits, the novel RRAM-based ones have the following advantages. First, the simple structure, high operating speed, low energy consumption, and excellent endurance of RRAMs enable related logic circuits with higher integration density and better performance. Second, the nonvolatility of RRAMs enables related logic circuits to store logic values and to perform logic operations simultaneously (i.e., ‘stateful’ logic circuits^7^), which can significantly reduce static power and may lead to instant boot^8^. The most basic and straightforward application of RRAMs for logic circuits is probably the construction of logic gates using RRAM cells. In initial studies, the construction of logic gates usually required more than one RRAM cell and other essential circuit elements such as resistors and capacitors, and the function of a given gate configuration was often exclusive^7^,^9^,^10^. For example, two RRAM cells and one resistor were adopted by Terabe *et al*.^10^ to construct both AND and OR gates, but the RRAM cells in these two gate configurations are just opposite in orientation. To simplify gate configuration and to realise multiple functions in a single gate configuration, single RRAM cells were subsequently introduced to implement logic operations^11^,^12^. For instance, Linn *et al*.^12^ demonstrated that 14 of 16 Boolean logic functions can be realised within a single bipolar resistive switch (BRS) or complementary resistive switch (CRS) in at most three sequential cycles, but the other two operations (i.e., XOR and XNOR functions) are theoretically exclusive within a single BRS or CRS. Recently, taking advantage of both positive and negative reading biases, You *et al*.^13^ reported the realisation of all 16 Boolean logic functions in a single switchable diode in three logic cycles. Although this study is indeed of great importance, switchable diode is very rare, and consequently its prospects for practical application are uncertain. Another approach to implementing all 16 Boolean logic functions is a three-terminal structure proposed very recently by Zhou *et al*.^14^, which will certainly face integration difficulty in crossbar logic circuits. In this study, we focus on the enormous potential of single CRS cells for logic applications. First, we introduce a method for constructing CRS cells with an intrinsic switchable diode and experimentally demonstrate the feasibility of implementing complete Boolean logic functions by utilising this diode. Second, we develop a method for implementing complete Boolean logic functions by utilising the complementary switching behaviour itself of any CRS cell and experimentally demonstrate its feasibility. These results, in particular the complementary switching behaviour itself-based complete Boolean logic functions with natural immunity to the sneak-path issue of crossbar logic circuits^15^, are believed to be capable of significantly advancing the theoretical research and practical applications of RRAM-based logic circuits. Moreover, a method for tri-level storage is developed in CRS cells with an intrinsic switchable diode, which can act as an alternative technique for realising ultra-high-density storage.

## Results and Discussion

**Figure 1:**
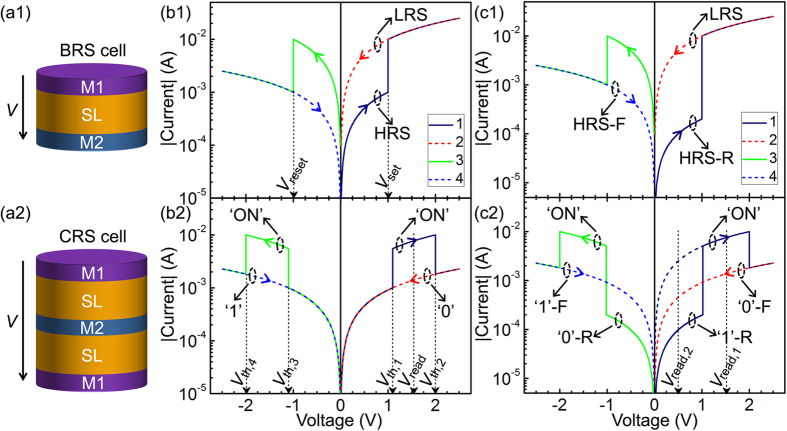
Simulated *I–V* characteristics of regular and peculiar CRS cells. Schematic device structures of (**a1**) a BRS cell and (**a2**) a corresponding CRS cell. M1, SL, and M2 represent metal electrode 1, active switching layer, and metal electrode 2, respectively. Simulated *I–V* characteristics of (**b1**) regular and (**c1**) peculiar BRS cells. Simulation parameters: LRS resistance (*R*_L_), 100 Ω; HRS resistance (*R*_H_), 1000 Ω; HRS-F resistance (*R*_H−F_), 1000 Ω; HRS-R resistance (*R*_H−R_), 5000 Ω; *V*_set_, 1 V; *V*_reset_, −1 V. Simulated *I–V* characteristics of (**b2**) regular and (**c2**) peculiar CRS cells based on the BRS *I–V* characteristics in (**b1**,**c1**), respectively. The navy dash line in (**c2**) is the linear extrapolation of ‘ON’ state. *V*_read_ and *V*_read,1_ are the read voltages for conventional bi-level storage application of CRS cells, and *V*_read,2_ is the read voltage for novel tri-level storage application of peculiar CRS cells.

The basic elements of CRS cells are BRS cells, which are composed of a simple tri-layer structure (Fig. 1a1) and can be reversibly switched between a high resistance state (HRS) and a low resistance state (LRS) under opposite voltage polarities (Fig. 1b1). The transition from the HRS to the LRS and the corresponding threshold voltage are denoted as the set process and *V*_set_, respectively, while their opposites are denoted as the reset process and *V*_reset_, respectively. After two identical BRS cells are anti-serially connected by a common electrode layer, a CRS cell that was pioneered by Linn *et al*.^15^ to solve the sneak-path issue of BRS-based crossbar arrays forms, as schematically shown in Fig. 1a2. There are three states in a complete switching cycle of CRS cells (Fig. 1b2): ‘1’ denotes the HRS/LRS (top BRS cell/bottom BRS cell) state, ‘ON’ denotes the LRS/LRS state, and ‘0’ denotes the LRS/HRS state. The threshold voltages for the transitions from ‘1’ to ‘ON’, from ‘ON’ to ‘0’, from ‘0’ to ‘ON’, and from ‘ON’ to ‘1’ are denoted as *V*_th,1_, *V*_th,2_, *V*_th,3_, and *V*_th,4_, respectively, as depicted in Fig. 1b2. The *V*_read_ in this figure represents the voltage for the read operation. For the standard CRS operation, ‘1’ and ‘0’ are used as memory states, and ‘ON’ is used to distinguish ‘1’ from ‘0’ in a destructive read operation. This is because ‘1’ and ‘0’ of common CRS cells with the *I*–*V* characteristic similar to Fig. 1b2 cannot be distinguished under a non-destructive voltage between *V*_th,3_ and *V*_th,1_ 15,16,17,18,19. These CRS cells are denoted as regular cells in the current study, and the related switching behaviours are denoted as regular complementary switching behaviours. Given that the *I–V* characteristics of ‘1’ and ‘0’ are dominated by the BRS cells in HRS and that the BRS cells in HRS for ‘1’ and ‘0’ are opposite in orientation, we suppose that ‘1’ and ‘0’ can be distinguished under a non-destructive voltage in CRS cells composed of BRS cells with a rectifying HRS. Figure 1c1 show the simulated *I–V* characteristics of such a BRS cell and the corresponding CRS cell, respectively. The postfixes ‘-F’ and ‘-R’ in these figures are used to denote the forward and reverse directions of the rectifying states, respectively. Indeed, ‘1’ and ‘0’ can be evidently distinguished under a non-destructive voltage, such as the *V*_read,2_ in Fig. 1c2, thus theoretically supporting the correctness of our supposition. Such CRS cells are denoted as peculiar cells in the current study, and related switching behaviours are denoted as peculiar complementary switching behaviours. Compared with regular CRS cells, the apparent and straightforward advantage of peculiar cells is the feasibility of using not only ‘1’ and ‘0’ but also ‘ON’ as memory states, i.e., tri-level storage. More importantly, peculiar CRS cells have an intrinsic switchable diode, i.e., the hysteresis loop “‘1’-R → ‘0’-F → ‘0’-R → ‘1’-F → ‘1’-R → ···” shown in Fig. 1c2, thereby exhibiting the feasibility of implementing complete Boolean logic functions^13^. In addition, it is instructive to examine why the HRS-R is set to be the same as, rather than opposite to, the set polarity in Fig. 1c1. First, this phenomenon is in accordance with most of the reported BRS cells with a rectifying HRS, such as (Ti, Ta, W)/Ta_2_O_5_/Pt^20^,^21^, (Ti, TiN)/HfO_2_/Pt^22^,^23^, Cu/ZnO/(Pt, Pd)^24^,^25^, Ag/ZrO_2_/Pt^26^, and Cu/P3HT:PCBM/ITO^27^. Second, this phenomenon is essential to ensure that the intrinsic switchable diode in Fig. 1c2 has the same switching polarity as that in ref. 13.

**Figure 2:**
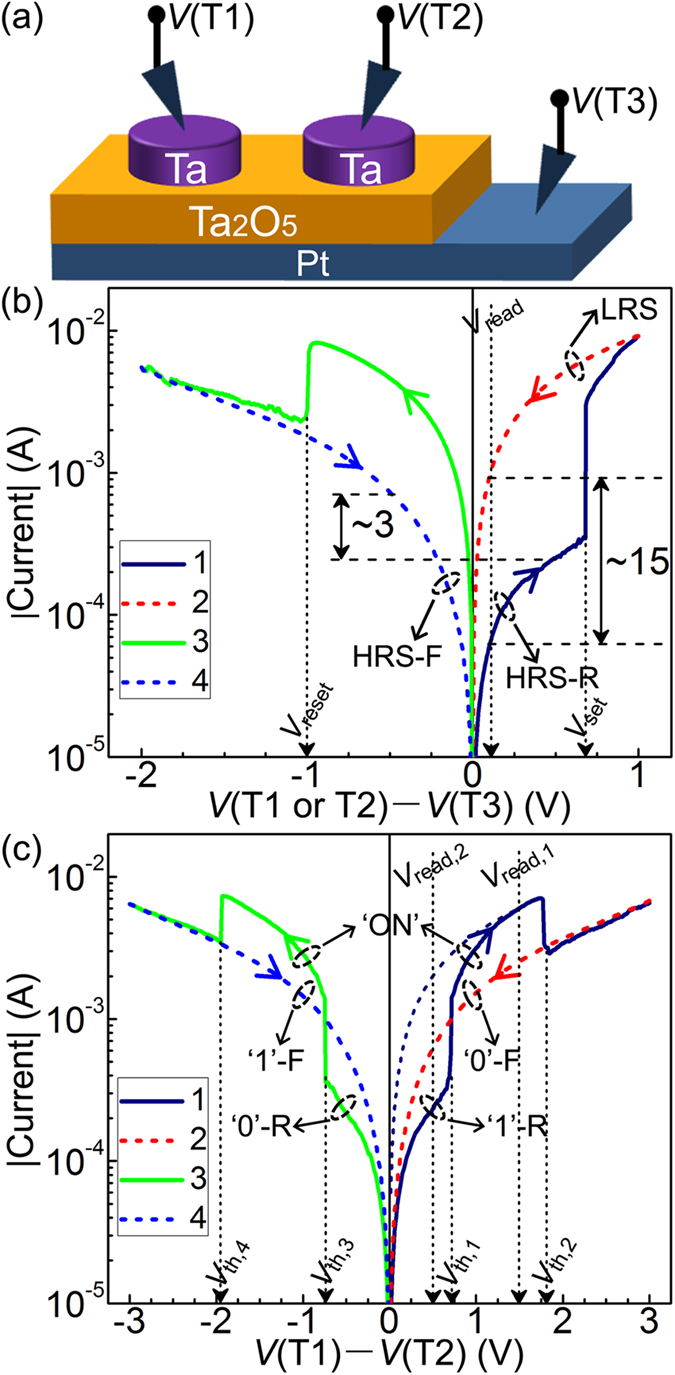
Experimental demonstration of peculiar BRS and corresponding CRS cells. (**a**) Schematic device structure and measurement configuration. (**b**) Measured *I–V* characteristic of Ta/Ta_2_O_5_/Pt BRS cells. Memory window: ~15@0.1 V; rectification ratio of HRS: ~3@ ± 0.5 V. (**c**) Measured *I–V* characteristic of Ta/Ta_2_O_5_/Pt/Ta_2_O_5_/Ta CRS cells. It reveals evidently an intrinsic switchable diode that can be used for tri-level storage and for implementing complete Boolean logic functions. The navy dash line is the linear extrapolation of ‘ON’ state at 1.5 V. *V*_read,1_ and *V*_read,2_ are the read voltages for conventional bi-level storage and for novel tri-level storage, respectively.

To experimentally demonstrate the correctness of our supposition concerning peculiar CRS cells, Ta_2_O_5_-based peculiar BRS and corresponding CRS cells were fabricated and systematically characterised. The choice of Ta_2_O_5_ as the active switching layer is deliberate, because Ta_2_O_5_-based RRAMs exhibit an ultra-high operating speed of ~100 ps^28^, extreme endurance performance of >10^12^ switching cycles^29^, excellent retention property of >10 years at 85 °C^30^, and great versatility in switching behaviour^31^,^32^, consequently holding out promising prospects for commercialisation. The device structure and measurement configuration are schematically shown in Fig. 2a. For BRS measurement, an external voltage was applied between terminal 1 or terminal 2 (T1 or T2) and terminal 3 (T3). After an initial forming process at ~3 V (see Supplementary Fig. S1 online), bipolar switching behaviour with a positive set process (~0.7 V), a negative reset process (~−1 V), and a large memory window of ~15@0.1 V was observed, as can be seen in Fig. 2b, which is attributed to the electric field-induced formation/rupture of an oxygen vacancy filament^20^,^21^,^33^. More importantly, the HRS shows a rectifying behaviour similar to that shown in Fig. 1c1. The rectifying HRS has been acknowledged to originate from the Schottky barrier at the Ta_2_O_5_/Pt interface^34^, and the rectification ratio is estimated to be ~3@ ± 0.5 V. The switching behaviour in Fig. 2b has been demonstrated to show satisfactory switching uniformity and retention performance (see Supplementary Fig. S2 online), thus suggesting that the Ta/Ta_2_O_5_/Pt BRS cells are suitable for constructing peculiar CRS cells. A peculiar CRS cell can be easily obtained by applying an external voltage between T1 and T2 but keeping T3 floating, i.e., Ta/Ta_2_O_5_/Pt/Ta_2_O_5_/Ta CRS cell. Indeed, a peculiar complementary switching behaviour similar to that in Fig. 1c2 was observed, as shown in Fig. 2c, thereby experimentally supporting the correctness of our supposition concerning peculiar CRS cells. It should be noted that such switching behaviour has also been observed in ref. 29, which further corroborates our supposition. To examine the feasibility of tri-level storage, a six-step voltage sweep method (see Supplementary Fig. S3 online) was adopted. With this method, 200 successive switching cycles were performed and then statistically analysed. The obtained results (see Supplementary Fig. S4 online) clearly show a gap not only between ‘1’-R resistance (*R*_‘1’-R_) and ‘0’-F resistance (*R*_‘0’-F_) but also between *R*_‘0’-F_ and ‘ON’ resistance (*R*_‘ON’_), thus supporting the feasibility of tri-level storage and demonstrating satisfactory switching uniformity in Ta/Ta_2_O_5_/Pt/Ta_2_O_5_/Ta CRS cells. Here, it should be pointed out that (i) when peculiar CRS cells are used for tri-level storage, additional nonlinear access devices are needed to settle the sneak-path issue of related crossbar arrays; (ii) compared with the approach for multilevel storage using regular complementary switching behaviours^35^,^36^, the main advantage of our approach is the elimination of the troublesome destructive read operation.

**Figure 3:**
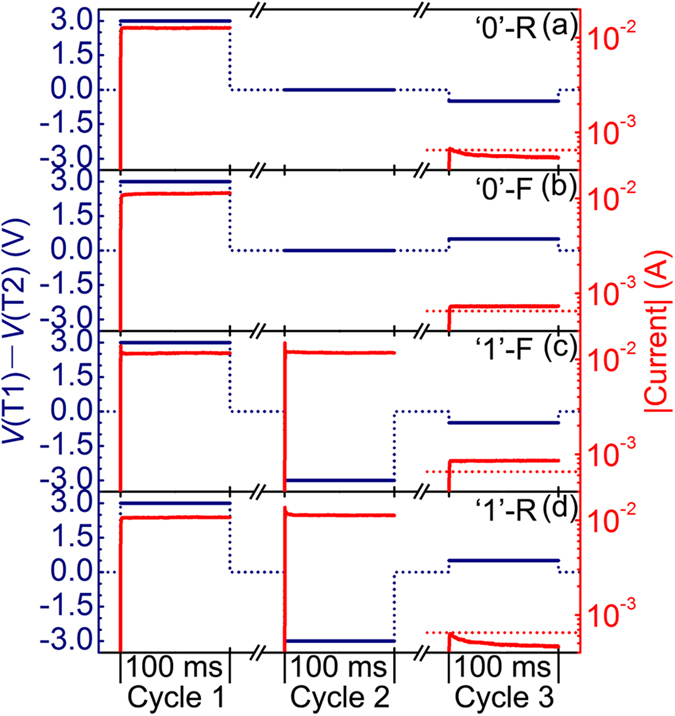
Experimental results of tri-cycle logic operation based on the intrinsic switchable diode of a Ta/Ta_2_O_5_/Pt/Ta_2_O_5_/Ta CRS cell. (**a**) Cycle 1, T1 − T2 = 1; cycle 2, T1 − T2 = 0; cycle 3, *r* = 0; output = 0. (**b**) Cycle 1, T1 − T2 = 1; cycle 2, T1 − T2 = 0; cycle 3, *r* = 1; output = 1. (**c**) Cycle 1, T1 − T2 = 1; cycle 2, T1 − T2 = −1; cycle 3, *r* = 0; output = 1. (**d**) Cycle 1, T1 − T2 = 1; cycle 2, T1 − T2 = −1; cycle 3, *r* = 1; output = 0. The navy solid curves represent the potential difference between T1 and T2 in each logic cycle, while the navy dot ones serve as a guide to the eye. The red solid curves represent the measured current in each logic cycle, while the red dot ones serve as the threshold current level for output = 0 (lower) and output = 1 (higher). The physical states responsible for the measured current in cycle 3 are clearly marked.

We now demonstrate the feasibility of implementing complete Boolean logic functions on the basis of the observed intrinsic switchable diode in Fig. 2c. The implementation of each logic operation needs three logic cycles, including two write cycles (cycles 1 and 2) with ±3 V and one read cycle (cycle 3) with ±0.5 V. For write cycles, the input logic variables *p* and *q* are used to represent the voltage potential values of T1 and T2. Assuming logic 1 for high potential (3 V) and logic 0 for low potential (0 V), the potential difference between T1 and T2 can be 3 V (T1 − T2 = 1), or 0 V (T1 − T2 = 0), or −3 V (T1 − T2 = −1). In contrast, for the read cycle, the input logic variables *p* and *q* are used to directly represent the potential difference between T1 and T2, i.e., the reading bias (*r*). A positive reading bias (0.5 V) is linked to *r* = 1, and a negative reading bias (−0.5 V) is linked to *r* = 0. The output of each logic operation is determined by the measured current in the read cycle (i.e., cycle 3). ‘0’-F and ‘1’-F will lead to a higher absolute read current and are assigned to logic 1, whereas ‘0’-R and ‘1’-R will lead to a lower absolute read current and are assigned to logic 0. For a more detailed description of this tri-cycle method for logic operation, readers are suggested to refer to ref. 13. Given that single BRS and CRS cells are considered to be capable of implementing all Boolean logic functions except XOR and XNOR operations and that switchable diodes are more talented than BRS and CRS cells in logic applications^12^,^13^, the success in implementing XOR and XNOR operations is enough to support the feasibility of implementing complete Boolean logic functions. The combination of Fig. 3 and Table 1 shows the obtained experimental results, which undoubtedly demonstrates the success in implementing XOR and XNOR operations. For simplicity, we take only the XOR operation as an example to provide a detailed description. In cycle 1, the 3 V pulse wrote the device into ‘0’. In cycle 2, with T1 assigned to 0 and T2 assigned to the input variable *q* (Table 1), the device remained at ‘0’ with *q* = 0 (Fig. 3a,b) and was transformed into ‘1’ with *q* = 1 (Fig. 3c,d). In cycle 3, with *r* assigned to *p*, ‘0’ was read as ‘0’-R with *p* = 0 (i.e., output = *p* XOR *q* = 0 XOR 0 = 0, Fig. 3a) and as ‘0’-F with *p* = 1 (i.e., output = *p* XOR *q* = 1 XOR 0 = 1, Fig. 3b), and ‘1’ was read as ‘1’-F with *p* = 0 (i.e., output = *p* XOR *q* = 0 XOR 1 = 1, Fig. 3c) and as ‘1’-R with *p* = 1(i.e., output = *p* XOR *q* = 1 XOR 1 = 0, Fig. 3d). Therefore, these results have definitely demonstrated the feasibility of implementing complete Boolean logic functions based on the intrinsic switchable diode of a single peculiar CRS cell.

**Figure 4:**
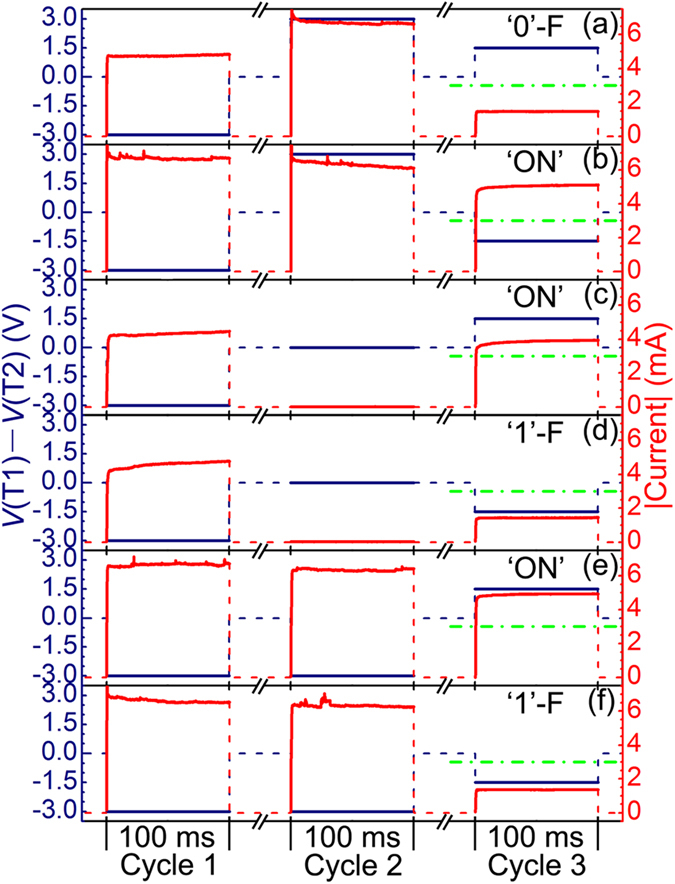
Experimental results of tri-cycle logic operation based on the complementary switching behavior itself, rather than the intrinsic switchable diode, of a Ta/Ta_2_O_5_/Pt/Ta_2_O_5_/Ta CRS cell. (**a**) Cycle 1, T1 − T2 = −1; cycle 2, T1 − T2 = 1; cycle 3, *r* = 1; output = 0. (**b**) Cycle 1, T1 − T2 = −1; cycle 2, T1 − T2 = 1; cycle 3, *r* = 0; output = 1. (**c**) Cycle 1, T1 − T2 = −1; cycle 2, T1 − T2 = 0; cycle 3, *r* = 1; output = 1. (**d**) Cycle 1, T1 − T2 = −1; cycle 2, T1 − T2 = 0; cycle 3, *r* = 0; output = 0. (**e**) Cycle 1, T1 − T2 = −1; cycle 2, T1 − T2 = −1; cycle 3, *r* = 1; output = 1. (**f**) Cycle 1, T1 − T2 = −1; cycle 2, T1 − T2 = −1; cycle 3, *r* = 0; output = 0. The navy and red solid curves represent the potential difference between T1 and T2 and the measured current in each logic cycle, respectively, while the navy and red dash ones both serve as a guide to the eye. The green dash dot curves serve as the threshold current level for output = 0 (lower) and output = 1 (higher). The physical states responsible for the measured current in cycle 3 are clearly marked.

There is no doubt that the intrinsic switchable diodes can significantly advance related CRS cells for logic applications. It has to be admitted, however, that only a portion of the reported BRS cells are suitable for constructing CRS cells with an intrinsic switchable diode. Inspired by the method of switchable diodes for logic applications, we herein develop a method for implementing complete Boolean logic functions in any CRS cell on the basis of the complementary switching behaviour itself. The theoretical basis of this method is that the complementary switching behaviour itself is in fact a ‘destructive’ switchable diode. This can be understood on the basis of the regular complementary switching behaviour in Fig. 1b2 as follows. For state ‘1’, −*V*_read_ and *V*_read_ will lead to lower and higher read currents, respectively, whereas for state ‘0’, −*V*_read_ and *V*_read_ will lead to higher and lower read currents, respectively. Hence, states ‘1’ and ‘0’ can be regarded as diodes with positive and negative polarities as forward directions, respectively. The word ‘destructive’ refers to the fact that the forward read operations of both states are destructive because both sates will be transformed into state ‘ON’. Similar to that for intrinsic switchable diodes, the logic operation method for these ‘destructive’ switchable diodes is also tri-cycle. The main difference between them is the reading bias in cycle 3, which is non-destructive for the former diodes (i.e., *V*_th,3_ < *V*_read_ < *V*_th,1_) but is destructive for the latter diodes (i.e., *V*_th,4_ < *V*_read_ < *V*_th,3_ or *V*_th,1_ < *V*_read_ < *V*_th,2_). For the Ta/Ta_2_O_5_/Pt/Ta_2_O_5_/Ta CRS cells with the *I–V* characteristic in Fig. 2c, the destructive reading bias in cycle 3 is set to ±1.5 V. That is, the positive destructive reading bias (1.5 V) is linked to *r* = 1, and the negative destructive reading bias (−1.5 V) is linked to *r* = 0. The detailed method for implementing complete Boolean logic functions based on the complementary switching behaviour itself of Ta/Ta_2_O_5_/Pt/Ta_2_O_5_/Ta CRS cells is given in Table 2, and the corresponding experimental results are shown in Fig. 4. Once again, we take the XOR operation as an example to provide a detailed description. In cycle 1, the −3 V pulse wrote the device into ‘1’. In cycle 2, with T1 assigned to the input variable *q* and T2 assigned to 0 (Table 2), the device remained at ‘1’ with *q* = 0 (Fig. 4c,d) and was transformed into ‘0’ with *q* = 1 (Fig. 4a,b). In cycle 3, with *r* assigned to *p*, ‘1’ was read as ‘1’-F with *p* = 0 (i.e., output = *p* XOR *q* = 0 XOR 0 = 0, Fig. 4d) and as ‘ON’ with *p* = 1 (i.e., output = *p* XOR *q* = 1 XOR 0 = 1, Fig. 4c), and ‘0’ was read as ‘ON’ with *p* = 0 (i.e., output = *p* XOR *q* = 0 XOR 1 = 1, Fig. 4b) and as ‘0’-F with *p* = 1 (i.e., output = *p* XOR *q* = 1 XOR 1 = 0, Fig. 4a). Thus, these results have conclusively demonstrated the feasibility of implementing complete Boolean logic functions on the basis of the complementary switching behavior itself of any CRS cell. Of course, for regular CRS cells, the ‘0’-F in Fig. 4a and ‘1’-F in Fig. 4d,f should be changed to ‘0’ and ‘1’, respectively. Besides, three points are noteworthy here. First, owing to the destructive read operation for output = 1, a write-back step is needed in practical applications after such a read operation. Second, the complementary switching behavior itself-based complete Boolean logic functions are naturally immune to the sneak-path issue of crossbar logic circuits^15^. Third, after the submission of our study for publication, Siemon *et al*.^37^ published an independent study in which they also proposed a method for implementing complete Boolean logic functions in any CRS cell on the basis of the complementary switching behaviour itself. The method proposed by Siemon *et al*. and that in our study are indeed very similar because their physical basis is identical, and the main difference between the two methods lies in the read operation in cycle 3, i.e., the spike read scheme in their method and the level read scheme in ours.

Before concluding, it is instructive to present a discussion, from the circuitry point of view, of the application prospects of our proposed methods for implementing complete Boolean logic functions in a single CRS cell. Above all, our methods have advanced significantly the practicability of single RRAM device-based Boolean logic functions. Therefore, more complex functions such as the adder can be more easily realised, given RRAMs’ ease of integration^2^,^3^. Second, our methods can make the peculiar CRS cell-based logic circuits versatile. The intrinsic switchable diode-based method is non-destructive in read cycles and therefore has a higher operating speed, whereas the complementary switching behaviour itself-based method is naturally immune to the sneak-path issue and consequently has superior reliability. Hence, the former and the latter methods can be intentionally selected for the cases with emphases on operating speed and on reliability, respectively, leading to a better performance for peculiar CRS cell-based logic circuits. Finally, it should be admitted that, owing to the conditional read operation needed by some logic functions such as XOR, our methods will inevitably cause an increase in complexity of the peripheral control circuits. However, given the significant simplification in the core logic circuits achieved by our methods, such an increase in peripheral circuitry overhead might be acceptable in most cases.

In summary, the huge potential of single CRS cells for logic applications has been theoretically analysed and then experimentally studied on the basis of the Ta/Ta_2_O_5_/Pt/Ta_2_O_5_/Ta CRS cells that exhibit satisfactory switching uniformity. It is found that BRS cells with a rectifying HRS can be used for constructing peculiar CRS cells with an intrinsic switchable diode and that the intrinsic switchable diode of a single peculiar CRS cell is robust enough to realise the complete Boolean logic functions. More importantly, a method for implementing complete Boolean logic functions based on the complementary switching behaviour itself of any single CRS cell has been discovered and experimentally demonstrated. These results, in particular the complementary switching behaviour itself-based complete Boolean logic functions with natural immunity to the sneak-path issue of crossbar logic circuits, are believed to be capable of significantly advancing both our understanding and commercialisation of RRAM-based logic circuits. Moreover, the constructed peculiar CRS cells with an intrinsic switchable diode are demonstrated to be feasible for tri-level storage, which can serve as an alternative method of realising ultra-high-density data storage.

## Methods

The Ta/Ta_2_O_5_/Pt BRS cells were fabricated at room temperature and on a commercial Pt(~120 nm)/Ti(~15 nm)/SiO_2_/Si substrate. The ~120 nm Pt layer served as the common bottom electrode for all Ta/Ta_2_O_5_/Pt BRS cells on this substrate. Prior to device fabrication, the substrate was ultrasonically cleaned in sequence in acetone, ethanol, and deionized water (8 min for each solvent). Subsequently, the blanket Ta_2_O_5_ layer (~10 nm in thickness) was deposited by radio-frequency (RF) magnetron sputtering (60 W) with a ceramic Ta_2_O_5_ target in a pure argon atmosphere (~0.4 Pa). Finally, the isolated Ta top electrodes (~60 nm in thickness and 50 μm in diameter) were patterned by the conventional ultraviolet lithography and lift-off processes, and were deposited by direct current (DC) magnetron sputtering in a pure argon atmosphere (~0.35 Pa). All electrical measurements were conducted on a semiconductor device analyzer (B1500A, Agilent) in atmospheric environment and at room temperature. For BRS measurement, the external voltage was applied between a Ta top electrode and the common Pt bottom electrode, while for CRS measurement, the external voltage was applied between two Ta top electrodes with the common Pt bottom electrode floating, i.e., constructing Ta/Ta_2_O_5_/Pt/Ta_2_O_5_/Ta CRS cells.

## Additional Information

**How to cite this article**: Gao, S. *et al*. Implementation of Complete Boolean Logic Functions in Single Complementary Resistive Switch. *Sci. Rep*. **5**, 15467; doi: 10.1038/srep15467 (2015).

## Supplementary Material

Supplementary Information

## Figures and Tables

**Table 1:** The truth table of XOR and XNOR operations and corresponding experimental results in Fig. 3.

Logic operation	Input		Output	Cycle 1			Cycle 2			Cycle 3	Experimental result
	*p*	*q*		T1	T2	T1 − T2	T1	T2	T1 − T2	*r*	
*p* XOR *q*	0	0	0	1	0	1	0	*q*	0	*p* = 0	Fig. 3a
	1	0	1	1	0	1	0	*q*	0	*p* = 1	Fig. 3b
	0	1	1	1	0	1	0	*q*	−1	*p* = 0	Fig. 3c
	1	1	0	1	0	1	0	*q*	−1	*p* = 1	Fig. 3d
*p* XNOR *q*	0	0	1	1	0	1	0	*q*	0	¬*p* = 1	Fig. 3b
	1	0	0	1	0	1	0	*q*	0	¬*p* = 0	Fig. 3a
	0	1	0	1	0	1	0	*q*	−1	¬*p* = 1	Fig. 3d
	1	1	1	1	0	1	0	*q*	−1	¬*p* = 0	Fig. 3c

‘¬*p*’ in this table represents the NOT *p* operation, i.e., ¬*p* (*p* = 1) = 0 and ¬*p* (*p* = 0) = 1.

**Table 2:** The truth table of complete Boolean logic functions and corresponding experimental results in Fig. 4.

Logic operation	Input		Output	Cycle 1			Cycle 2			Cycle 3	Experimental result
	*p*	*q*		T1	T2	T1 − T2	T1	T2	T1 − T2	*r*	
True	0	0	1	0	1	−1	*p*	0	0	¬*p* = 1	Fig. 4c
	1	0	1	0	1	−1	*p*	0	1	¬*p* = 0	Fig. 4b
	0	1	1	0	1	−1	*p*	0	0	¬*p* = 1	Fig. 4c
	1	1	1	0	1	−1	*p*	0	1	¬*p* = 0	Fig. 4b
False	0	0	0	0	1	−1	*p*	0	0	*p* = 0	Fig. 4d
	1	0	0	0	1	−1	*p*	0	1	*p* = 1	Fig. 4a
	0	1	0	0	1	−1	*p*	0	0	*p* = 0	Fig. 4d
	1	1	0	0	1	−1	*p*	0	1	*p* = 1	Fig. 4a
*p*	0	0	0	0	1	−1	1	*p*	1	1	Fig. 4a
	1	0	1	0	1	−1	1	*p*	0	1	Fig. 4c
	0	1	0	0	1	−1	1	*p*	1	1	Fig. 4a
	1	1	1	0	1	−1	1	*p*	0	1	Fig. 4c
*q*	0	0	0	0	1	−1	1	*q*	1	1	Fig. 4a
	1	0	0	0	1	−1	1	*q*	1	1	Fig. 4a
	0	1	1	0	1	−1	1	*q*	0	1	Fig. 4c
	1	1	1	0	1	−1	1	*q*	0	1	Fig. 4c
NOT *p*	0	0	1	0	1	−1	1	*p*	1	0	Fig. 4b
	1	0	0	0	1	−1	1	*p*	0	0	Fig. 4d
	0	1	1	0	1	−1	1	*p*	1	0	Fig. 4b
	1	1	0	0	1	−1	1	*p*	0	0	Fig. 4d
NOT *q*	0	0	1	0	1	−1	1	*q*	1	0	Fig. 4b
	1	0	1	0	1	−1	1	*q*	1	0	Fig. 4b
	0	1	0	0	1	−1	1	*q*	0	0	Fig. 4d
	1	1	0	0	1	−1	1	*q*	0	0	Fig. 4d
*p* AND *q*	0	0	0	0	1	−1	*p*	*q*	0	*p* = 0	Fig. 4d
	1	0	0	0	1	−1	*p*	*q*	1	*p* = 1	Fig. 4a
	0	1	0	0	1	−1	*p*	*q*	−1	*p* = 0	Fig. 4f
	1	1	1	0	1	−1	*p*	*q*	0	*p* = 1	Fig. 4c
*p* NAND *q*	0	0	1	0	1	−1	*p*	*q*	0	¬*p* = 1	Fig. 4c
	1	0	1	0	1	−1	*p*	*q*	1	¬*p* = 0	Fig. 4b
	0	1	1	0	1	−1	*p*	*q*	−1	¬*p* = 1	Fig. 4e
	1	1	0	0	1	−1	*p*	*q*	0	¬*p* = 0	Fig. 4d
*p* OR *q*	0	0	0	0	1	−1	*q*	*p*	0	*p* = 0	Fig. 4d
	1	0	1	0	1	−1	*q*	*p*	−1	*p* = 1	Fig. 4e
	0	1	1	0	1	−1	*q*	*p*	1	*p* = 0	Fig. 4b
	1	1	1	0	1	−1	*q*	*p*	0	*p* = 1	Fig. 4c
*p* NOR *q*	0	0	1	0	1	−1	*q*	*p*	0	¬*p* = 1	Fig. 4c
	1	0	0	0	1	−1	*q*	*p*	−1	¬*p* = 0	Fig. 4f
	0	1	0	0	1	−1	*q*	*p*	1	¬*p* = 1	Fig. 4a
	1	1	0	0	1	−1	*q*	*p*	0	¬*p* = 0	Fig. 4d
*p* IMP *q*	0	0	1	0	1	−1	*p*	*q*	0	1	Fig. 4c
	1	0	0	0	1	−1	*p*	*q*	1	1	Fig. 4a
	0	1	1	0	1	−1	*p*	*q*	−1	1	Fig. 4e
	1	1	1	0	1	−1	*p*	*q*	0	1	Fig. 4c
*p* NIMP *q*	0	0	0	0	1	−1	*p*	*q*	0	0	Fig. 4d
	1	0	1	0	1	−1	*p*	*q*	1	0	Fig. 4b
	0	1	0	0	1	−1	*p*	*q*	−1	0	Fig. 4f
	1	1	0	0	1	−1	*p*	*q*	0	0	Fig. 4d
*p* RIMP *q*	0	0	1	0	1	−1	*q*	*p*	0	1	Fig. 4c
	1	0	1	0	1	−1	*q*	*p*	−1	1	Fig. 4e
	0	1	0	0	1	−1	*q*	*p*	1	1	Fig. 4a
	1	1	1	0	1	−1	*q*	*p*	0	1	Fig. 4c
*p* RNIMP *q*	0	0	0	0	1	−1	*q*	*p*	0	0	Fig. 4d
	1	0	0	0	1	−1	*q*	*p*	−1	0	Fig. 4f
	0	1	1	0	1	−1	*q*	*p*	1	0	Fig. 4b
	1	1	0	0	1	−1	*q*	*p*	0	0	Fig. 4d
*p* XOR *q*	0	0	0	0	1	−1	*q*	0	0	*p* = 0	Fig. 4d
	1	0	1	0	1	−1	*q*	0	0	*p* = 1	Fig. 4c
	0	1	1	0	1	−1	*q*	0	1	*p* = 0	Fig. 4b
	1	1	0	0	1	−1	*q*	0	1	*p* = 1	Fig. 4a
*p* XNOR *q*	0	0	1	0	1	−1	*q*	0	0	¬*p* = 1	Fig. 4c
	1	0	0	0	1	−1	*q*	0	0	¬*p* = 0	Fig. 4d
	0	1	0	0	1	−1	*q*	0	1	¬*p* = 1	Fig. 4a
	1	1	1	0	1	−1	*q*	0	1	¬*p* = 0	Fig. 4b

‘¬*p*’ in this table represents the NOT *p* operation, i.e., ¬*p* (*p* = 1) = 0 and ¬*p* (*p* = 0) = 1.

## References

[b1] International Technology Roadmap for Semiconductors, 2013 Edition, (2014) Available at: http://www.itrs.net/ITRS%201999-2014%20Mtgs,%20Presentations%20&%20Links/2013ITRS/Summary2013.htm (Date of access: 06/06/2015).

[b2] Pan F., Gao S., Chen C., Song C. & Zeng F. Recent progress in resistive random access memories: Materials, switching mechanisms, and performance. Mater. Sci. Eng. R 83, 1–59 (2014).

[b3] Seok J. Y. . A Review of Three-Dimensional Resistive Switching Cross-Bar Array Memories from the Integration and Materials Property Points of View. Adv. Funct. Mater. 24, 5316–5339 (2014).

[b4] Yang J. J., Strukov D. B. & Stewart D. R. Memristive devices for computing. Nat. Nanotechnol. 8, 13–24 (2013). 23269430 10.1038/nnano.2012.240

[b5] Waser R., Dittmann R., Staikov G. & Szot K. Redox-based resistive switching memories nanoionic mechanisms, prospects, and challenges. Adv. Mater. 21, 2632–2663 (2009). 10.1002/adma.200900375 36751064

[b6] Sawa A. Resistive switching in transition metal oxides. Mater. Today 11, 28–36 (2008).

[b7] Borghetti J. . ‘Memristive’ switches enable ‘stateful’ logic operations via material implication. Nature 464, 873–876 (2010). 20376145 10.1038/nature08940

[b8] Balatti S., Ambrogio S. & Ielmini D. Normally-off Logic Based on Resistive Switches—Part I: Logic Gates. IEEE Trans. Electron Dev. 62, 1831–1838 (2015).

[b9] Kuekes P. J., Stewart D. R. & Williams R. S. The crossbar latch: Logic value storage, restoration, and inversion in crossbar circuits. J. Appl. Phys. 97, 034301 (2005).

[b10] Terabe K., Hasegawa T., Nakayama T. & Aono M. Quantized conductance atomic switch. Nature 433, 47–50 (2005). 15635405 10.1038/nature03190

[b11] Rosezin R., Linn E., Kügeler C., Bruchhaus R. & Waser R. Crossbar Logic Using Bipolar and Complementary Resistive Switches. IEEE Electron Dev. Lett. 32, 710–712 (2011).

[b12] Linn E., Rosezin R., Tappertzhofen S., Böttger U. & Waser R. Beyond von Neumann—logic operations in passive crossbar arrays alongside memory operations. Nanotechnology 23, 305205 (2012). 22782173 10.1088/0957-4484/23/30/305205

[b13] You T. . Exploiting Memristive BiFeO_3_ Bilayer Structures for Compact Sequential Logics. Adv. Funct. Mater. 24, 3357–3365 (2014).

[b14] Zhou Y., Li Y., Xu L., Zhong S., Sun H. & Miao, X. 16 Boolean logics in three steps with two anti-serially connected memristors. Appl. Phys. Lett. 106, 233502 (2015).

[b15] Linn E., Rosezin R., Kügeler C. & Waser R. Complementary Resistive Switches for Passive Nanocrossbar Memories. Nat. Mater. 9, 403–406 (2010). 20400954 10.1038/nmat2748

[b16] Rosezin R. . Integrated Complementary Resistive Switches for Passive High-Density Nanocrossbar Arrays. IEEE Electron Dev. Lett. 32, 191–193 (2011).

[b17] Bae Y. C. . Oxygen Ion Drift-Induced Complementary Resistive Switching in Homo TiO_*x*_/TiO_*y*_/TiO_*x*_ and Hetero TiO_*x*_/TiON/TiO_*x*_ Triple Multilayer Frameworks. Adv. Funct. Mater. 22, 709–716 (2012).

[b18] Chen C. . Effect of Electrode Materials on AlN-Based Bipolar and Complementary Resistive Switching. ACS Appl. Mater. Interfaces 5, 1793–1799 (2013). 23422310 10.1021/am303128h

[b19] van den Hurk J., Havel V., Linn E., Waser R. & Valov I. Ag/GeS_*x*_/Pt-based complementary resistive switches for hybrid CMOS/Nanoelectronic logic and memory architectures. Sci. Rep. 3, 2856 (2013). 24091355 10.1038/srep02856 PMC3790209

[b20] Chen C. . Conductance quantization in oxygen-anion-migration-based resistive switching memory devices. Appl. Phys. Lett. 103, 043510 (2013).

[b21] Kim K. M. . Low Variability Resistor–Memristor Circuit Masking the Actual Memristor States. Adv. Electron. Mater. 1, 1500095 (2015).

[b22] Chen Y. S. . Well controlled multiple resistive switching states in the Al local doped HfO_2_ resistive random access memory device. J. Appl. Phys. 113, 164507 (2013).

[b23] Fang Z. . Current Conduction Model for Oxide-Based Resistive Random Access Memory Verified by Low-Frequency Noise Analysis. IEEE Trans. Electron Dev. 60, 1272–1275 (2013).

[b24] Zhuge F. . Improvement of resistive switching in Cu/ZnO/Pt sandwiches by weakening the randomicity of the formation/rupture of Cu filaments. Nanotechnology 22, 275204 (2011). 21613680 10.1088/0957-4484/22/27/275204

[b25] Yang Y. . Nonvolatile resistive switching in single crystalline ZnO nanowires. Nanoscale 3, 1917–1921 (2011). 21394361 10.1039/c1nr10096c

[b26] Liu Q. . Controllable Growth of Nanoscale Conductive Filaments in Solid-Electrolyte-Based ReRAM by Using a Metal Nanocrystal Covered Bottom Electrode. ACS Nano 4, 6162–6168 (2010). 20853865 10.1021/nn1017582

[b27] Gao S., Song C., Chen C., Zeng F. & Pan F. Dynamic Processes of Resistive Switching in Metallic Filament-Based Organic Memory Devices. J. Phys. Chem. C 116, 17955–17959 (2012).

[b28] Torrezan A. C., Strachan J. P., Medeiros-Ribeiro G. & Williams R. S. Sub-nanosecond switching of a tantalum oxide memristor. Nanotechnology 22, 485203 (2011). 22071289 10.1088/0957-4484/22/48/485203

[b29] Lee M. J. . A fast, high-endurance and scalable non-volatile memory device made from asymmetric Ta_2_O_5−*x*_/TaO_2−*x*_ bilayer structures. Nat. Mater. 10, 625–630 (2011). 21743450 10.1038/nmat3070

[b30] Choi S., Lee J., Kim S. & Lu W. D. Retention failure analysis of metal-oxide based resistive memory. Appl. Phys. Lett. 105, 113510 (2014).

[b31] Yang Y., Choi S. & Lu W. Oxide Heterostructure Resistive Memory. Nano Lett. 13, 2908–2915 (2013). 23724783 10.1021/nl401287w

[b32] Gao S. . Tuning the switching behavior of binary oxide-based resistive memory devices by inserting anultra-thin chemically active metal nanolayer: a case study on the Ta_2_O_5_–Ta system. Phys. Chem. Chem. Phys. 17, 12849–12856 (2015). 25907552 10.1039/c5cp01235j

[b33] Park G.-S. . *In situ* observation of filamentary conducting channels in an asymmetric Ta_2_O_5−*x*_/TaO_2−*x*_ bilayer structure. Nat. Commun. 4, 2382 (2013). 24008898 10.1038/ncomms3382

[b34] Hur J. H. . Modeling for bipolar resistive memory switching in transition-metal oxides. Phys. Rev. B 82, 155321 (2010).

[b35] Balatti S., Larentis S., Gilmer D. C. & Ielmini D. Multiple Memory States in Resistive Switching Devices Through Controlled Size and Orientation of the Conductive Filament. Adv. Mater. 25, 1474–1478 (2013). 23288623 10.1002/adma.201204097

[b36] Schönhals A., Waser R., Menzel S. & Rana V. 3-bit Read Scheme for Single Layer Ta_2_O_5_ ReRAM. Proceedings of Non-Volatile Memory Technology Symposium 2014, p. 1 4.

[b37] Siemon A. . Realization of Boolean Logic Functionality Using Redox-Based Memristive Devices. Adv. Funct. Mater. 10.1002/adfm.201500865 (2015).

