# Supplementary material for: Implementation of Complete Boolean Logic Functions in Single Complementary Resistive Switch
Source: Sci Rep. 2015 Oct 21;5:15467. doi: 10.1038/srep15467 (PMC4614354; doi:10.1038/srep15467)
Supplement: Supplementary Information [file srep15467-s1.doc]

Supplementary Information

Implementation of Complete Boolean Logic Functions in Single Complementary Resistive Switch

Shuang Gao, Fei Zeng,* Minjuan Wang, Guangyue Wang, Cheng Song & Feng Pan*

Key Laboratory of Advanced Materials (MOE), School of Materials Science and Engineering, Tsinghua University, Beijing, 100084, China

Correspondence and requests for materials should be addressed to F.Z. (email: zengfei@mail.tsinghua.edu.cn) or F.P. (email: panf@mail.tsinghua.edu.cn).

**S1. Initial forming process of the Ta/Ta_2_O_5_/Pt BRS cells**


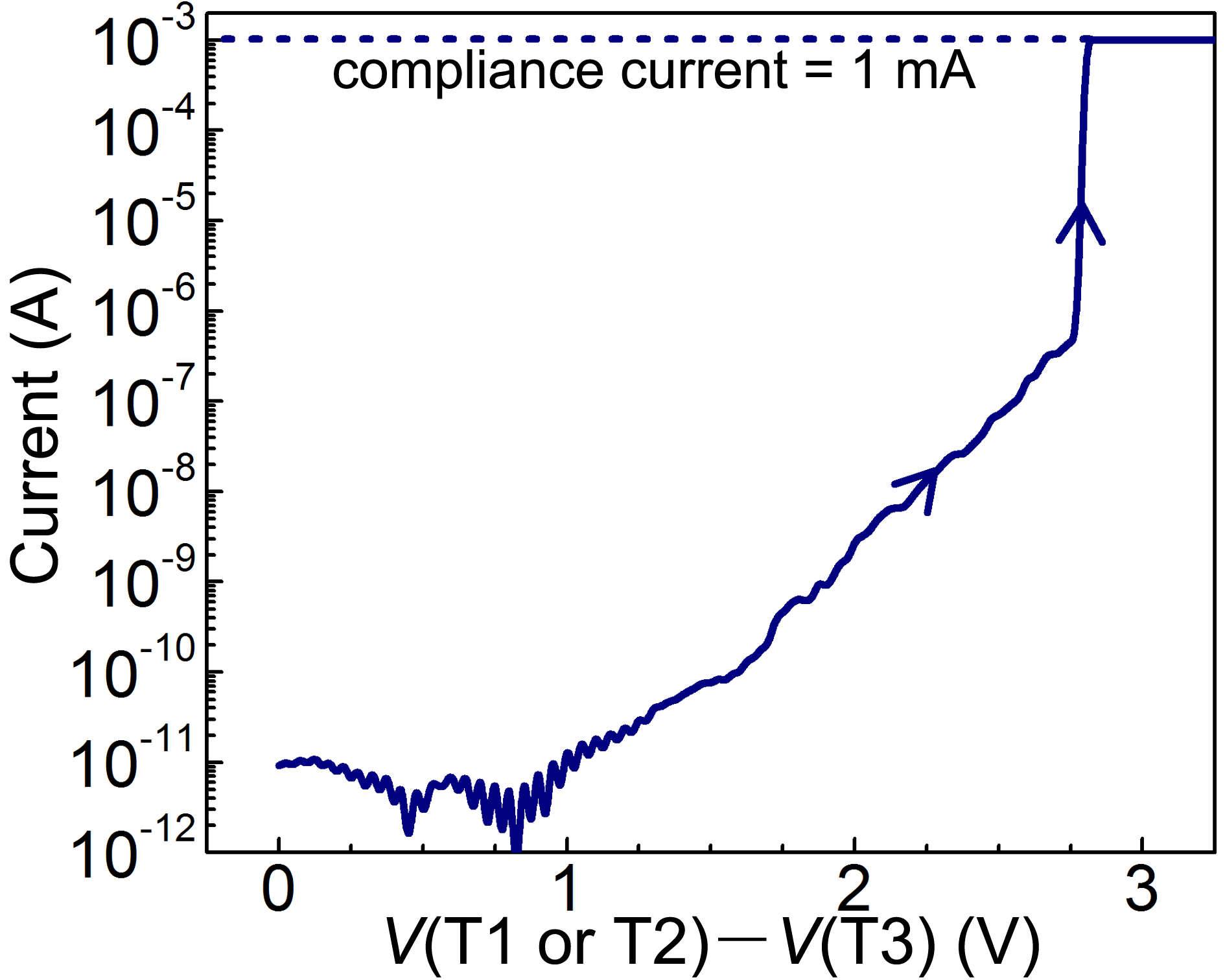


**Supplementary Figure S1.** Initial forming process of the Ta/Ta_2_O_5_/Pt BRS cells. The compliance current of 1 mA was adopted to prevent permanent breakdown.

**S2. Switching uniformity and retention performance of the Ta/Ta_2_O_5_/Pt BRS cells**

The cumulative probabilities of critical switching parameters in Fig. S2b and c are obtained based on the *I–V* curves of successive 200 switching cycles in Fig. S2a. One can see that both the distributions of *V*_set_ and *V*_reset_ in Fig. S2b and that of *R*_L_@0.1 V, *R*_H_@0.1 V, *R*_H_@0.5 V, and *R*_H_@–0.5 V in Fig. S2c are very tight. More importantly, these is clear gap between the distributions of *R*_H_@0.5 V and *R*_H_@–0.5 V in Fig. S2c. These results together reveal satisfactory switching uniformity of the Ta/Ta_2_O_5_/Pt BRS cells. In Fig. S2d, there is no degradation in both *R*_L_ and *R*_H_ after a time period of 1 000 s and under a constant voltate stress of 0.1 V, thus suggesting satisfactory retention performance of the Ta/Ta_2_O_5_/Pt BRS cells.


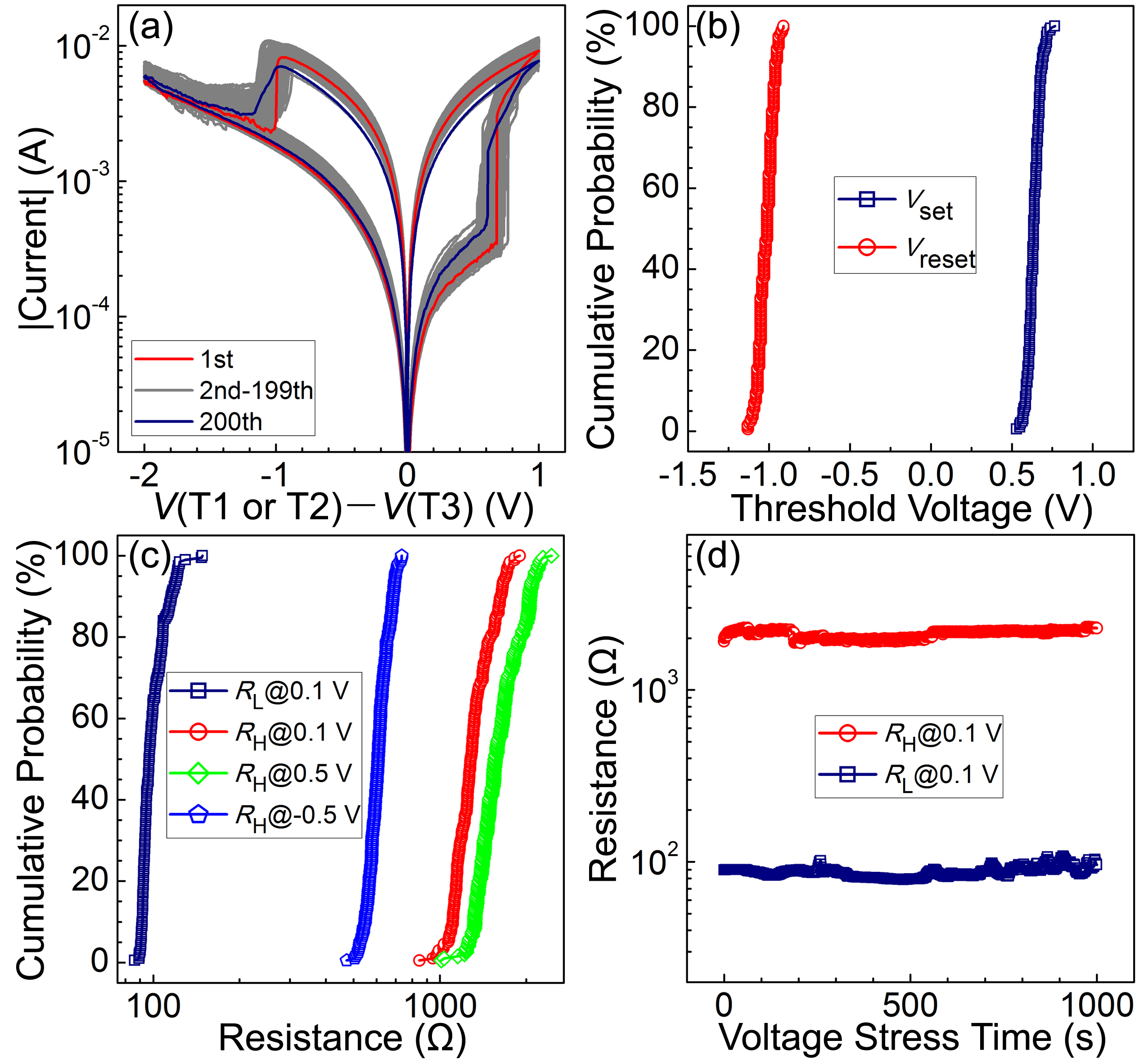


**Supplementary Figure S2.** Switching uniformity and retention performance of the Ta/Ta_2_O_5_/Pt BRS cells. (a) *I–V* curves of successive 200 switching cycles. (b) Cumulative probabilities of *V*_set_ and *V*_reset_. (c) Cumulative probabilities of *R*_L_@0.1 V, *R*_H_@0.1 V, *R*_H_@0.5 V, and *R*_H_@–0.5 V. (d) Retention property under a constant voltate stress of 0.1 V.

**S3. The six-step voltage sweep method for tri-level storage in the Ta/Ta_2_O_5_/Pt/Ta_2_O_5_/Ta CRS cells**


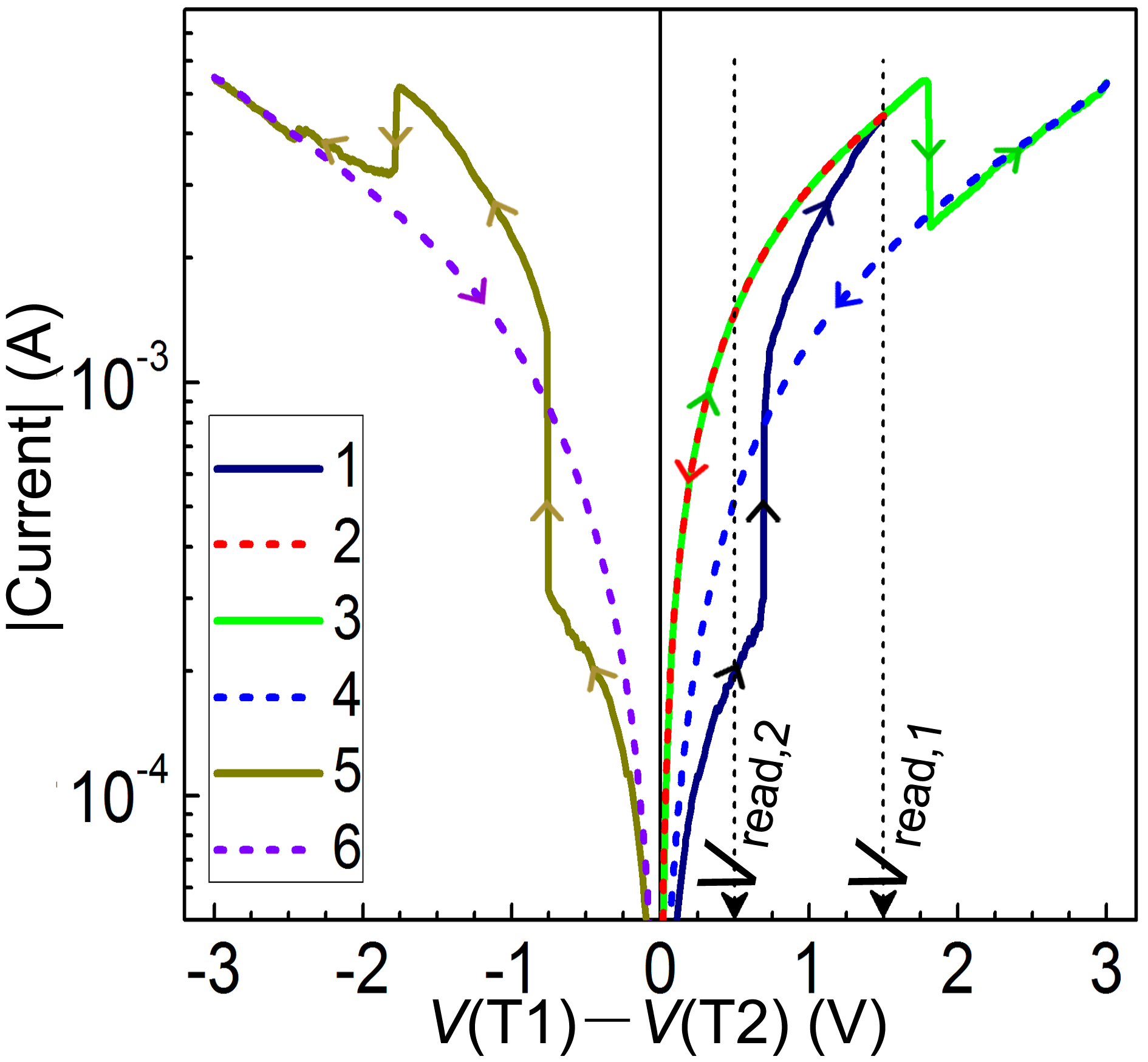


**Supplementary Figure S3.** The six-step voltage sweep method for tri-level storage in the Ta/Ta_2_O_5_/Pt/Ta_2_O_5_/Ta CRS cells. The data for conventional bi-level storage can be extracted at *V*_read,1_ = 1.5 V, while that for novel tri-level storage can be extracted at *V*_read,2_ = 0.5 V.

**S4. Tri-level storage and switching uniformity of the Ta/Ta_2_O_5_/Pt/Ta_2_O_5_/Ta CRS cells**

The cumulative probabilities of critical switching parameters in Fig. S4b–d are obtained based on the *I–V* curves of successive 200 switching cycles in Fig. S4a. Figure S4b shows the cumulative probabilities of thresholdvoltages including *V*_th,1_, *V*_th,2_, *V*_th,3_, and *V*_th,4_. The cumulative probabilities of resistance values for conventional bi-level storage and for novel tri-level storage are provided in Fig. S4c and d, respectively. These results revel that the distributions of all examined switching parameters are very tight, thus suggesting satisfactory switching uniformity of the Ta/Ta_2_O_5_/Pt/Ta_2_O_5_/Ta CRS cells. In Fig. S4c, *R*_‘1’_@1.5 V and *R*_‘0’_@1.5 V are the read resistances of ‘1’ and ‘0’ at *V*_read,1_ = 1.5 V, respectively, while *R*_‘1’_@0.5 V and *R*_‘0’_@0.5 V are the leakage resistances of ‘1’ and ‘0’ at 1/3*V*_read,1_ = 0.5 V, respectively. One can easily see that *R*_‘1’_@0.5 V and *R*_‘0’_@0.5 V are both larger than *R*_‘0’_@1.5 V, thus suppressing effectively the sneak-path issue of crossbar arrays. In Fig. S4d, *R*_‘ON’_@0.5 V, *R*_‘0’_@0.5 V, and *R*_‘1’_@0.5 V correspond to *R*_‘ON’_, *R*_‘0’-F_, *R*_‘1’-R_ in the main text, respectively. Due to the very tight distributions of *R*_‘ON’_@0.5 V, *R*_‘0’_@0.5 V, and *R*_‘1’_@0.5 V, a clearly gap exists not only between *R*_‘1’_@0.5 V and *R*_‘0’_@0.5 V but also between *R*_‘0’_@0.5 V and *R*_‘ON’_@0.5 V, thus supporting the feasibility for tri-level storage of the Ta/Ta_2_O_5_/Pt/Ta_2_O_5_/Ta CRS cells.


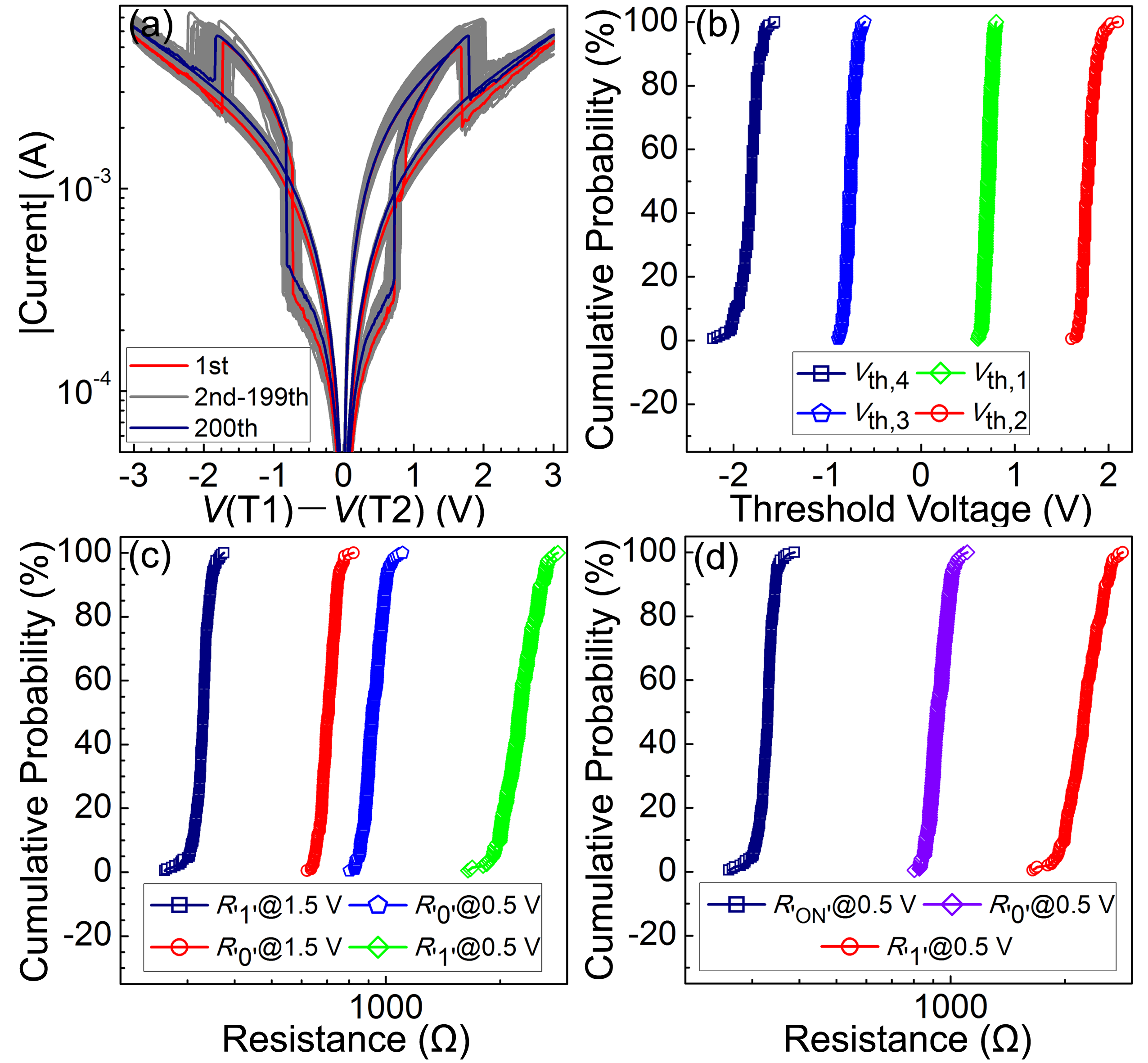


**Supplementary Figure S4.** Tri-level storage and switching uniformity of the Ta/Ta_2_O_5_/Pt/Ta_2_O_5_/Ta CRS cells. (a) *I–V* curves of successive 200 switching cycles. (b) Cumulative probabilities of *V*_th,1_, *V*_th,2_, *V*_th,3_, and *V*_th,4_. (c) Cumulative probabilities of *R*_‘1’_@1.5 V, *R*_‘0’_@1.5 V, *R*_‘0’_@0.5 V, and *R*_‘1’_@0.5 V. (d) Cumulative probabilities of *R*_‘ON’_@0.5 V, *R*_‘0’_@0.5 V, and *R*_‘1’_@0.5 V.
